# A link between social isolation during the coronavirus outbreak and social alignment in balcony parties

**DOI:** 10.1371/journal.pone.0264109

**Published:** 2022-04-06

**Authors:** Hila Z. Gvirts Problovski, Mor Sherman, Victoria Melnikova

**Affiliations:** Department of Behavioral Sciences and Psychology, Ariel University, Ari’el, Israel; University of Padova, ITALY

## Abstract

With the ongoing COVID-19 pandemic, there is a growing need for assessing the psychological costs of social isolation (SI). We examine whether the balcony party during the first outbreak of the pandemic is associated with how individuals cope with SI as well as its causes and consequences during the COVID-19 outbreak. A total of 303 quarantined persons responded to a Web-based survey. We found that the effect of balcony parties on the psychological costs of SI is dependent on the self-reported levels of SI. Those who experienced high levels of causes of SI perceived the balcony parties as more beneficial in inducing positive affect and reducing negative affect in comparison to those who experienced low levels of causes of SI. The opposite pattern was observed when individuals were asked about their participation in these parties: individuals with high levels of consequences of SI experienced balcony parties as less beneficial than similar pre-outbreak gathering events, while individuals with low levels of consequences of SI showed an opposite pattern. Finally, for those with high levels of causes of SI and consequences of SI, balcony parties did not meet the expectation of creating feelings of communal solidarity. However, a discrepancy between high SI expectations and experience was not found for those with low SI. Our findings demonstrate that the balcony parties are beneficial in reducing the emotional cost of social isolation–but only for those who feel low levels of SI. The fact that individuals with high levels of SI expected more out of these parties suggests the need to develop interventions aimed at optimizing their expectations. As society enters a new period in which the costs of social distancing may be higher, our findings are valuable for understanding the psychological battle that individuals face while in social isolation.

## Introduction

In December 2019, the first case of the novel Coronavirus (also known as COVID-19) was reported in Wuhan, China. Since then, the virus has been spread rapidly throughout Asia, Europe, the Middle East, and the Americas. On March 11 2020, it was declared a global pandemic by the World Health Organization [1]. Currently, no vaccine exists for treatment of the virus. As a result, health professionals have prescribed social distancing and quarantine as means to reduce viral spreading [2]. Social distancing refers to efforts to minimize close interpersonal interactions in public. As individuals may be contagious and unidentified as virus carriers, social distancing reduces the risk of virus transmission [3]. Quarantine can be defined as movement restriction of persons who presumed to have been exposed to a contagious disease [4]. In battling COVID-19, many countries used social distancing and quarantine to impose major movement restrictions on large populations in specific at-risk zones.

However, the emotional and psychological costs of social distancing and quarantine should be weighed against their obvious public health benefits. Indeed, the emotional and psychological impact of COVID-19 is still largely unknown.

A recent review points to a series psychological hardships both during and after imposition of quarantine in previous disease outbreaks [5]. The authors conclude that people under quarantined generally reported high prevalence of psychological distress symptoms. In addition, longer durations of quarantine were associated with poorer mental health. Wang et al. [6] surveyed the situation in China during the first two weeks of the COVID-19 outbreak. They showed that even at this very early stage of the outbreak respondents rated the psychological impact as moderate-to-severe. Notably, the ripple effects of the quarantine strategy included exacerbation of social isolation and loneliness. Loneliness can be defined as distress resulting from the perception that one’s social relationships are quantitatively less than desirable [7–9]. Social isolation refers to an objective lack of interpersonal interactions or engagement with one’s community [10]. The notion that social isolation leads to loneliness has gained support from studies that found a positive association between low frequency of social contact and loneliness [11, 12]. However, others found a low correlation between loneliness and social isolation [13–15], suggesting the possibility that social isolation does not always leads to loneliness. One possible explanation for this discrepancy is the fact that social isolation can be defined objectively, whereas loneliness is a subjective feeling that may not necessarily emerge from social isolation. Indeed, this distinction may explain why associations between the two are inconsistently reported across studies [16]. Here, we focus on the question of what can be done to mitigate subjective feelings of loneliness that may emerge due to “stay-at-home” regulations widely implemented in many countries.

In fact, quarantine and social isolation during COVID-19 has resulted in novel forms of self-expression–many documented in videos from a variety of countries such as Italy, Spain, and Israel. For example, citizens around the world can be seen clapping, singing, and playing musical instruments or applauding healthcare workers–all from their apartment balconies. Imber‐Black [17] have recently introduced the term "balcony party" as a ritual invented during this period in which individuals opened their windows or came out on their small balconies or on rooftops to play musical instruments, sing, clap hands, bang pots and pans. This type of social gathering includes a heterogeneous set of activities that occurs during social gatherings and should thus be examined as a tool to break the walls of social isolation.

Of course, technology offers plenty of platforms for people to stay in touch with each other. Nevertheless, technology-mediated communication lacks the naturalistic element that characterizes everyday face-to-face social interaction. For example, in the latter, interactants inhabit and share the same “physical space”. However, online meetings may be social but lack shared physical space [18]. From this perspective, balcony parties can be distinguished as social events from alternative technology-mediated forms of gathering (e.g. video chat).

Humans are a social species, attracted to interpersonal interaction and public gatherings. From a spatial attraction perspective, people often seek to share the same physical space and reduce distance between neighbors [19]. We may therefore hypothesize that balcony parties offer a possibility to share the same physical space while ensuring social distancing.

Under certain conditions, individuals automatically coordinate their behaviors with those of other participants in social interaction [20–22]. Therefore, it is not surprising that during balcony parties individuals align their behaviors with the behaviors of their neighbors through hand clapping, singing, or dancing. Notably, this behavioral alignment has been found to elicit reward sensations, encouraging closeness and connectedness [23]. Taken together, it is reasonable to assume that balcony parties facilitate naturalistic social interaction. Rewarding sensations associated with this type of interaction may thus act to mitigate the emotional costs of social isolation.

This research focuses on whether balcony parties are perceived and experienced as a tool for mitigating the psychological costs of social isolation. As noted above, the balcony party offers a more real-life and naturalistic form of social interaction compared to its technology-mediated counterpart. Notably, it may elicit social alignment which is defined as the tendency of individuals to align their motions, emotions, and cognitions [23]. In its basic form, social alignment refers to interpersonal motor synchrony—that is an overlap of movements between two or more people in time [24]. Interpersonal synchrony may occur spontaneously and unconsciously during everyday social interactions such as when our footsteps unconsciously synchronize with our partner’s while walking together or when we clap our hands in the same rhythm with others [25, 26]. Here, we propose that interpersonal synchrony (e.g. clapping hands) during COVID social gatherings can be viewed as an attempt to bring people together not only in space but also in time. Moreover, since interpersonal synchrony is a crucial component in facilitating social connection, we argue that these social gatherings also encouraged closeness and connectedness. Accordingly, we hypothesized that the more people feel socially isolated the more they will perceive and experience the balcony parties as beneficial in decreasing the costs of social isolation.

## Method

### Participants

All persons who were placed in quarantine/social isolation during the first coronavirus outbreaks in Italy and Israel were eligible for participation in this study. Information on the study and invitations to participate were posted on social media, in Hebrew and Italian. This study was approved by the Ethical Review Committee of Ariel University (Ariel, Israel). Written informed consent was obtained from the respondents before the administration of the questionnaire. The inclusion criteria included an age of more than 18 years and an agreement to participate in the survey. Participation was rewarded with a coupon distributed to the 68 student participants from Ariel University (included in the population of Israeli participants).

The first part of the survey was completed by 303 respondents, who were placed in quarantine or social isolation in Italy (n = 108) or Israel (n = 195) between March 23th, 2020 and April 2th, 2020. All respondents completed the first part of the survey. Table 1 reports the distributions of demographic characteristics of the respondents for the first part of the survey. As shown in Table 1, the median duration of quarantine was 14 days (interquartile range, 7–20 days). About 81.2% of respondents were female, 18–73 years of age, with 33.3% married and 63.4% with a college level of education or higher.

**Table 1:** The distributions of demographic characteristics of the respondents to the first part of the survey.

**SI**	**Total**	**Low SI**	**High SI**	**Statistical analyses**
		**N = 180**	**N = 123**	
Parametric measures [mean ± S.D.]				
Age	34.015±13.844	33.914±13.653	34.163±14.1748	U = 10757.5 p = .676
Days in Quarantine	14.60 ± 7.839	13.94±7.506	15.57±8.248	U = 9920.5, p = .124
Non-parametric measures [Number (%)]				
Gender (male/female)*	57 (18.8%), 246 (81.2%)	44 (24.4%), 136 (75.6%)	13 (10.6%), 110 (89.4%)	X^2^ = 9.211, p = .002
Education (Middle School, High school, Bacelor, Master, PhD or higher)	12 (4.0%), 99(32.7%), 79 (26.1%), 105(34.7%), 8(2.6%)	6(3.3%)’53(29.4%),51(28.3%), 63(35%), 7(4%)	6 (4.9%), 46(37.4%), 28 (22.8%), 42(40%), 1 (0.8%)	X^2^ = 5.358, p = .252
Family Status (marriage/single)	101 (33.3%),202 (66.7%)	59 (32.8%), 121 (67.2%)	42(34.1%), 81 (65.9%)	X^2^ = .062, p = .804
How many children in the family (no children,1 children, 2–4 children, more then 4)	226(74.6%), 32(10.6%), 44 (14.5%),1(0.3%)	134 (74.9%), 17 (9.5%), 29 (16.1%),0	92(74.8%),15 (12.2%), 15 (12.2%),1(0.3%)	X^2^ = 2.760, p = .430
Country(Italy/Israel)	108(35.6), 195(64,4%)	64(35.6%), 116(64.4%)	44(35.8%), 79(64.2%)	X^2^ = .001, p = .969
**Consequences of SI**	**total**	**Low levels of consequences**	**High levels of consequences**	**Statistical analyses**
		**N = 106**	**N = 197**	
Parametric measures [mean±S.D]				
Age	34.015±13.844	32.742±13.742	34.701±13.948	U = 9688.5,P = .300
Days in Quarantine*	14.6±7.839	12.59±7.301	15.69±7.922	U = 8065.5,P = .001
Non-parametric measure [number(%)]				
Gender (male/female)*	57(18.8%), 246(81.2%)	29(27.4%), 77(72.6%)	28(14.2%), 169(85.7%)	X^2^ = 7.797, p = .005
Education (Middle School, High school, Bacelor, Master, PhD or higher)*	12(4%),99(32.7%), 79(26.1%),105(34.7%), 8(2.6%)	0,36(34%), 33(31.1%), 32(30.2%), 5(4.7%)	12(6.1%),63(32%), 46(23.4%),73(37.1%), 3(1.5%)	X^2^ = 11.741, p = .019
Family Status (marriage/single)	101(33.3%), 202(66.7%)	31(29.2%), 75(70.8%)	70(35.5%),127(64.5%)	X^2^ = 1.226, p = .268
How many children in the family (no children,1 children, 2–4 children, more then 4)	226(74.6%), 32(10.6%), 44(14.5), 1(0.3%)	84(79.2%),9(8.5%), 13(12.3%),0	142(72.1%),23(11.7%), 31(15.7%), 1(0.5%)	X^2^ = 2.246, p = .523
Country(Italy/Israel)*	108(35.6%),195(64.4%)	29(27.4%), 77(72.6%)	79(40.1%), 118(59.9%)	X^2^ = 4.879, p = .027
**Causes of SI**	**Total**	**Low levels of causes**	**High levels causes**	**Statistical analyses**
		**N = 143**	**N = 157**	
Parametric measure [mean±S.D]				
Age	34.015±13.8	34.390±13.779	33.66±13.939	U = 10767,P = .362
Days in quarantine	14.6±7.839	14.54±7.584	33.66±8.092	U = 11403.5,P = .940
Non-parametric measure [number(%)]				
Gender (male/female)*	57(18.8%), 246(81.2%)	44(30.1%),102(69.9%)	13(8.3%), 144(91.7%)	X^2^ = 23.662, p = .000
Education (Middle School, High school, Bacelor, Master, PhD or higher)	12(4%), 99(32.7%), 79(26.1%), 105(34.7%), 8(2.6%)	4(2.7%), 46(31.5), 34(23.3%), 57(39%), 5(3.4%)	8(5.1%), 53(33.8%), 45(28.7%), 48(30.6%), 3(1.9%)	X^2^ = = 4.238, P = .375
Family Status (marriage/single)	101(33.3%), 202(66.7%)	53(36.3%), 93(63.7%)	48(30.6%), 109 (69.4%)	X^2^ = 1.117, p = .291
How many children in the family (no children,1 children, 2–4 children, more then 4)	226(74.6%), 32(10.6%), 44(14.5%), 1(0.3%)	107(73.3%),15(10.3),23(15.8%),1(0.7%)	119(75.8%), 17(10.8%), 21 (13.4%),0	X^2^ = 1.456, P = .693
Country(italy/israel)	108(35.6%), 195(64.4%)	60(41.1%),86(58.9%)	48(30.6%), 109(69.4%)	X^2^ = 3.652, p = .056

Note =

* p < .05/

The second part of the survey was completed by 211 (69.6%) respondents who participated at least once in a balcony party. Table 2 reports the distributions of demographic characteristics of the respondents for the second part of the survey. As shown in Table 2, about 85.8% of respondents were female, with 32.7% married and 57.3% with a college level of education or higher. On average, these respondents participated in 2.48 balcony parties.

**Table 2:** The distributions of demographic characteristics of the respondents for the second part of the survey.

**SI**	**Total**	**Low SI**	**High SI**	**Statistical analyses**
		**N = 117**	**N = 94**	
Parametric measures [mean ± S.D.]				
Age	32.346±13.391	32.082±13.236	32.689±13.658	U = 5325, p = .692
Days 6in Quarantine*	13.95 ± 7.279	12.83±6.989	15.41±7.426	U = 4438, p = .016
Non-parametric measures [Number (%)]				
Gender (male/female)	30(14.2%), 181(85.8%)	21(17.9%), 96(82.1%)	9(9.6%), 85(90.4%)	X^2^ = 2.997, p = .083
Education (Middle School, High school, Bacelor, Master, PhD or higher)	8(3.8%), 82(38.9%), 52(24.6%), 63(29.9%), 6(2.8%)	5(4.3%), 45(38.5%), 31(26.5%), 31(26.5%), 5(4.3%)	3(3.2%), 37(39.4%), 21(22.3%), 32(34%), 1(1.1%)	X^2^ = 3.420, p = .490
Family Status (marriage/single)	69(32.7%), 142(67.3%)	37(31.6%), 80(68.4%)	32(34%),62(66%)	X^2^ = .139, p = .710
How many children in the family (no children,1 children, 2–4 children, more then 4)	156(73.9%),23(10.9%), 31(14.7%),1(0.5%)	85(72.6%), 11(9.4%), 21(17.9%), 0	71(75.5%), 12(12.8%), 10(10.6%),1(1.1%)	X^2^ = 3.740, p = .291
Country(Italy/Israel)	58(27.5%) ,153(72.5%)	28(23.9%), 89(76.1%)	30(31.9%), 64(68.1%)	X^2^ = .1.667, p = .197
**Consequences of SI**	**Total**	**Low levels of consequences**	**High levels of consequences**	**Statistical analyses**
		**N = 74**	**N = 137**	
Parametric measures [mean±S.D]				
Age	32.346±13.391	30.995±13.454	33.098±13.484	U = 4428, P = .129
Days in Quarantine*	13.95 ± 7.279	11.89±6.919	15.10±7.246	U = 3776.5, P = .002
Non-parametric measure [number(%)]				
Gender (male/female)	30(14.2%), 181(85.8%)	15(20.3%), 59(79.7%)	15(10.9%), 122(89.1%)	X^2^ = 3.423, p = .064
Education (Middle School, High school, Bacelor, Master, PhD or higher)	8(3.9%),82(39.1%), 52(24.6%), 63(29.9%), 6(2.8%)	0, 33(44.6%), 19(25.7%), 18(24.3%), 4(5.4%)	8(6%), 49(35.8%), 33(24.1%), 45(32.8%), 2(1.5%)	X^2^ = 9.133, p = .058
Family Status (marriage/single)	69(32.7%), 142(67.3%)	20(27%), 54(73%)	49(35.8%)88(64.2%)	X^2^ = 1.667, p = .197
How many children in the family (no children,1 children, 2–4 children, more then 4)	156(73.9%), 23(11.6%), 31(14.7%),1(0.5%)	58(78.4%), 7(9.5%), 9(12.2%), 0	98(71.5%), 16(11.7%), 22(16.1%),1(0.7%)	X^2^ = 1.558, p = .669
Country(Italy/Israel)*	58(27.5%), 153(72.5%)	14(18.9%), 60(81.1%)	44(32.1%),93(67.9%)	X^2^ = 4.199, p = .040
**Causes of SI**	**Total**	**Low levels of causes**	**High levels causes**	**Statistical analyses**
		**N = 91**	**N = 120**	
Parametric measure [mean±S.D]				
Age	32.346±13.391	32.533±13.424	32.197±13.422	U = 5444,, P = .971
Days in quarantine	13.95 ± 7.279	13.5±7.181	14.31±7.367	U = 5065, P = .367
Non-parametric measure [number(%)]				
Gender (male/female)*	30(14.2%), 181(85.8%)	23(25.3%), 68(74.7%)	7(5.8%), 113(94.2%)	X^2^ = 16.038, p = .000
Education (Middle School, High school, Bacelor, Master, PhD or higher)	8(3.8%), 82(38.9%), 52(24.6%), 63(29.9%), 6(2.8%)	4(4.4%), 37(40.7%), 20(22%), 26(28.6%), 4(4.4%)	4(3.3%),45(37.5%), 32(26.7%), 37(30.8%), 2(1.7%)	X^2^ = 2.193, P = .778
Family Status (marriage/single)	69(32.7%), 142(67.3%)	31(34.1%), 60(65.9%)	38(31.7%), 82(68.3%)	X^2^ = .135, p = .713
How many children in the family (no children,1 children, 2–4 children, more then 4)	156(73.9%), 23(10.9%), 31(14.7%),1(0.5%)	66(72.5%), 10(11%), 14(15.4%),1(1.1%)	90(75%), 13(10.8%), 17(14.2%),0	X^2^ = 1.415, P = .709
Country(Italy/Israel)	58(27.5%), 153(72.5%)	27(29.7%), 64(70.3%)	31(25.8%), 89(74.2)	X^2^ = .382, p = .536

Note =

* p < .05

### Tasks, procedures and apparatus

#### Questionnaires

A web-based survey composed of both multiple choice and short-answer questions was completed by participants in quarantine/social isolation. The questionnaire was divided into two parts. The first part included items on: (1) demographic information, (2) the psychological impact of quarantine, and (3) attitudes towards balcony parties (see section 2.2.2). The second part included items on the beneficial outcomes of the balcony party. In this section, participants were asked to report their experience during the balcony party and similar gatherings that took place before the coronavirus outbreak. It took approximately 7 minutes to complete the first part of the questionnaire and 15 minutes to complete the second. While the first part was filled out by all subjects, 31.7% percent of the sample did not confirm participation in at least one balcony party and thus did not fill out the second part. S1 Table summarizes the demographic differences between those who participated in the first part of the survey and those who participated in the second part. The group of individuals who participated in at least one balcony party (and responded to two both of the survey) was significantly younger, less educated, had more females, reported less days in quarantine, and included more Israelis.

#### The first part of the survey

*Psychological impact of quarantine and social isolation*. The Psychological Impact questionnaire is a self-report measure designed to assess current subjective distress resulting from social isolation and quarantine, composed of five multiple-choice questions. Participants were instructed to read five statements and decide how much they either agree or disagree with each, using a Likert rating scale from 1 to 5. The five statements referred to their feelings while being socially isolated. They were asked to what degree they felt socially isolated, lonely, distress, longing for face-to-face social or naturalistic interaction with family/friends. The total Psychological Impact of quarantine and social isolation (SI) score ranged from 7 to 25. Additionally, for the total score, we created two other aspects of social isolation causes (i.e., reasons leading to social isolation) and consequences (i.e., feelings that stem from social distance). A correlation analysis was used to examine the relationships between different aspects of social isolation/quarantine. The data concerning the variables related to social isolation/quarantine were not normally distributed, as assessed by a Shapiro-Wilk test. Therefore, the correlation coefficients were calculated using Spearman’s correlation. Cronbach’s alpha for these five questions was .725.

*Attitude towards the balcony party*. To evaluate the attitudes of the general public towards balcony parties, nine multiple choice questions were asked, each with a Likert rating scale from 1 to 5. Participants were asked to rate how much they agreed with each of the following statements: (1) Balcony parties makes me feel united; (2) Balcony parties induce positive feeling; (3) People feel more supported by others; (4) People feel less lonely; (5) Balcony parties help in reducing social isolation; (6) Balcony parties helps distract people from their worries; (7) Balcony parties allow people to connect with their neighbors; (8) Balcony parties are good entertainment; and (9) Balcony parties help in reducing stress and anxiety. The maximum score is 45. The total attitude score ranged from 9 to 45. Cronbach’s alpha for these nine questions was .946.

#### The second part of the survey

*Beneficial outcomes of the balcony party*. To examine the beneficial outcomes of the balcony party, balcony parties were compared with similar pre-outbreak gatherings. As noted above, what balcony parties attempt to do is to bring people together in space and time. We, therefore, asked individuals about their experience of pre-COVID social gatherings in which they also align their motions in space and time (e.g. pre-COVID dancing at a party, dancing at a wedding, singing in a choir, etc.).

The questionnaire included seven multiple choice questions that were asked twice, each with a Likert rating scale from 1 to 5. One time the questions were asked in relation to beneficial outcomes of participating in balcony parties and one time in relation to pre-outbreak gatherings. Participants were asked to rate how much they agreed with each of the following statements: (1) The gathering makes me feel great! Singing/dancing makes me feel better; (2) The gathering makes us feel united and affiliated; (3) The gathering reduces the feeling of social isolation; (4) The gathering makes me feel less lonely; (5) The gathering makes me feel less anxious and distressed; (6) The gathering makes me feel more connected to my neighborhood; and (7) The gathering helps distract me from worries. The maximum score is 35 for each questionnaire. The total score for experience during the balcony party ranged from 11 to 35, whereas the total score for the pre-outbreak gathering experience ranged from 7 to 35. Cronbach’s alphas for questions on the balcony party was .877 and for pre-outbreak gatherings was .93.

### Statistical analysis

Analyses were conducted using SPSS, version 26. Note that in all the analyses we conducted, non-parametric tests were used due to the deviation of data from normality.

#### The first part of the survey

As shown in S2 Table, we found highly significant positive correlations between all of the five questions aimed at assessing attitude towards the balcony party. We, therefore, calculated an *index score* for social isolation (sum score of all five items). We then divided our sample into low and high Socially Isolated (SI) according to the median split. In addition, the five items were factor analyzed using principal component analysis (PCA) with Direct Oblimin rotation method to establish the underlying constructs. The analysis yielded two factors explaining 70.65% of the variance for the entire set of variables. Factor 1 explained 49.57% of the variance and was labeled consequences of SI and included the following items: (a) Since the corona outbreak I feel more socially isolated, (b) Since the corona outbreak I feel lonely, and (c) Since the corona outbreak I feel anxious. The second factor derived was labeled as the causes of these feelings (i.e. Causes of SI). The following items were loaded on the causes factor: (a) Since the corona outbreak I miss face-to-face social interaction and (b) Since the corona outbreak I miss naturalistic interaction with my friends/ family. The variance explained by this factor was 21.083%. The KMO (.655) and Bartlett’s Test of Sphericity both indicate that the set of variables are at least adequately related for factor analysis (*x*^2^ = 470.186, df = 10 p < .001). These findings suggest that we have identified two clear patterns of responses: one pattern of consequences and one of causes. These two tendencies are independent of one another. On the basis of these findings, participants were also classified into low and high levels of consequences of SI and low and high levels of causes of SI (according to the median split). As shown in Table 1, individuals with high SI, high levels of causes of SI and high levels of consequences of SI had a greater tendency to be female. Moreover, individuals with high levels of consequences of SI reported more days in quarantine, were more educated and had higher percentages of Italians. Consequently, these variables were added as covariates in the following analyses.

#### Attitude towards the balcony party

To determine whether individual differences in the self-reported levels of social isolation is associated with attitude towards the balcony parties, we conducted a multivariate covariance analysis of variance (MANCOVA) with gender as covariates and with SI group (low, high) as a between-subject factor. There were nine dependent measures (i.e. nine scores aimed at evaluating the attitude towards the balcony party, see above). Since factor analysis revealed two distinct factors, we were also interested in examining whether differences in causes of SI or consequences of SI were associated with attitude towards the balcony parties. To this end, we twice repeated the same analysis as described for SI group: once with causes of SI (low, high) as the between-subject factor and once with the consequences of SI (low, high) as the between-subject factor. In the later analysis, number of days in quarantine, education and country were added as additional covariates. The significant group effect was further explored using non-parametric tests (Mann–Whitney U test).

#### The second part of the survey

As in the first part of the survey, the group of participants who filled out the second part of the questionnaire were divided into low and high SI according to the median split. On the basis of the results of factor analysis (see above), which revealed two distinct factors (causes and consequences), participants were also classified into low and high levels of consequences of social isolation and low and high levels of causes of social isolation (according the median split). As shown in Table 2, individuals with high SI, high levels of causes of SI and high levels of consequences of SI reported more days in quarantine. Participants with high levels of consequences of SI had higher percentages of Italians, whereas participants with high levels of causes of SI had more females. Consequently, these variables were added as covariates in the following analyses.

#### Beneficial outcomes of participating in parties

To determine whether individual differences in the self-reported levels of social isolation are associated with beneficial outcomes of participating in balcony parties, we conducted 2 (high/low) by 2 (type of party) mixed design multivariate analysis of variance (MANCOVA) with days of quarantine as a covariant and with group (low/ high SI) as the between-subject factor, and with type of party (balcony/pre-outbreak parties) as the within-subject factor. There were seven dependent measures (i.e. each of the seven questions aimed at evaluating the beneficial outcomes of the two types of parties, see above). This analysis was repeated twice: once with causes of SI (low, high) as a between-subject factor and with gender as a covariant instead of days of quarantine and once with the consequences of SI (low, high) as the between-subject factor and with country (Israel/ Italy) as an additional covariant. The significant group effect was further explored using non-parametric tests (Mann–Whitney U test).

#### Gap between attitude and beneficial outcomes of balcony parties

To determine whether individual differences in the self-reported levels of social isolation are associated with the gap between attitude towards balcony parties and beneficial outcomes of participating in these parties, we conducted 2 (high/low SI) by 2 (attitude/expectation) mixed design multivariate analysis of variance (MANCOVA) with days of quarantine as a covariant, and with the two study groups (low SI, high SI) as the between-subject factor, and with type of measurements (attitude/expectation towards the balcony parties) as the within-subject factor. This analysis was repeated twice: once with causes of SI (low, high) as a between-subject factor and with gender as a covariant instead of days of quarantine and once with the consequences of SI (low, high) as the between-subject factor and with country (Israel/ Italy) as an additional covariant.

Wilcoxon tests with type of measurements (attitude/expectation towards the balcony parties) as the within-subject factor were subsequently conducted for each type of party separately to determine whether there were differences in the mean score of attitude towards balcony parties and beneficial outcomes of parties.

## Results

### Attitude towards the balcony party

#### Association between the self-reported levels of SI and attitude towards the balcony parties

**Fig 1:**
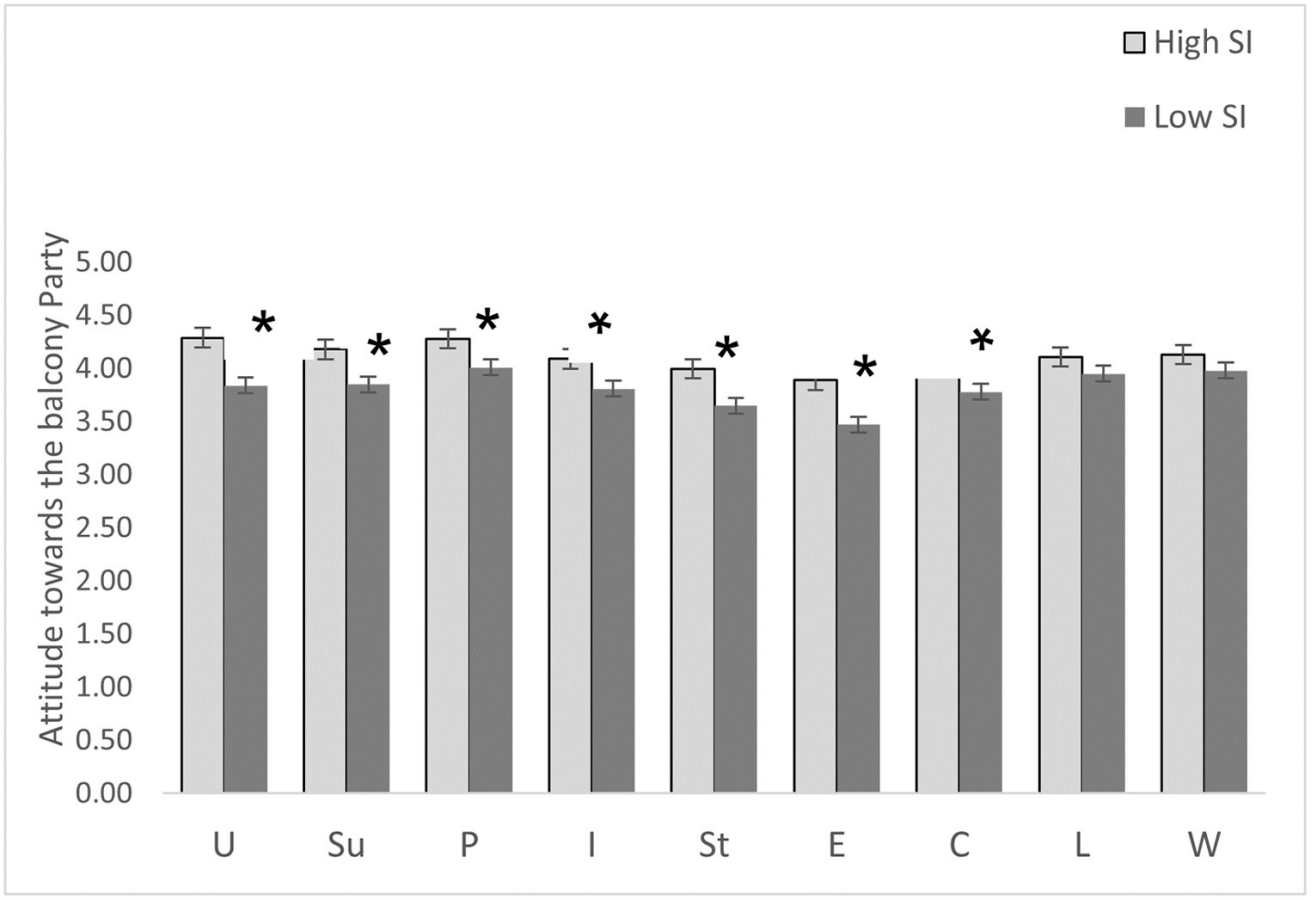
Average scores of attitude towards the balcony party divided into low and high SI groups. **U**–unity, **Su**-support, **P**-positive, **I**-isolation, **St**—stress, **E**-creating entertainment, **C**-connectedness, **L**-loneliness, **W**-worries. Note = * p < .05. The error bars represent the standard error.

The MANCOVA (with gender as covariant) revealed a significant group effect (f(9,286) = 2.526, p = .008), suggesting an association between the self-reported levels of SI and attitude towards the balcony parties. The self-reported levels of SI had a statistically significant group effect for balcony parties as inducing feelings of unity (1,294) = 14.611, p = .000), positivity (f(1,294) = 4.851, p = .028), support (f(1,294) = 7.451, p = .007) and connectedness (f(1,294) = 7.273, p = .007), and reduced isolation (f(1,294) = 3.975, p = .047) and stress (f(1,294) = 4.992, p = .026) as well as for creating entertainment (f(1,294) = 7.369, p = .007). However, the group effect was non-significant for the role of balcony parties in reducing feelings of loneliness (f(1,294) = 1.753, p = .187) and worries (f(1,294) = .980, p = .323). As shown in Fig 1, examination of the means indicated that individuals with higher levels of SI had a more positive attitude towards the balcony party.

#### Association between the self-reported levels of causes of SI and attitude towards the balcony parties

**Fig 2:**
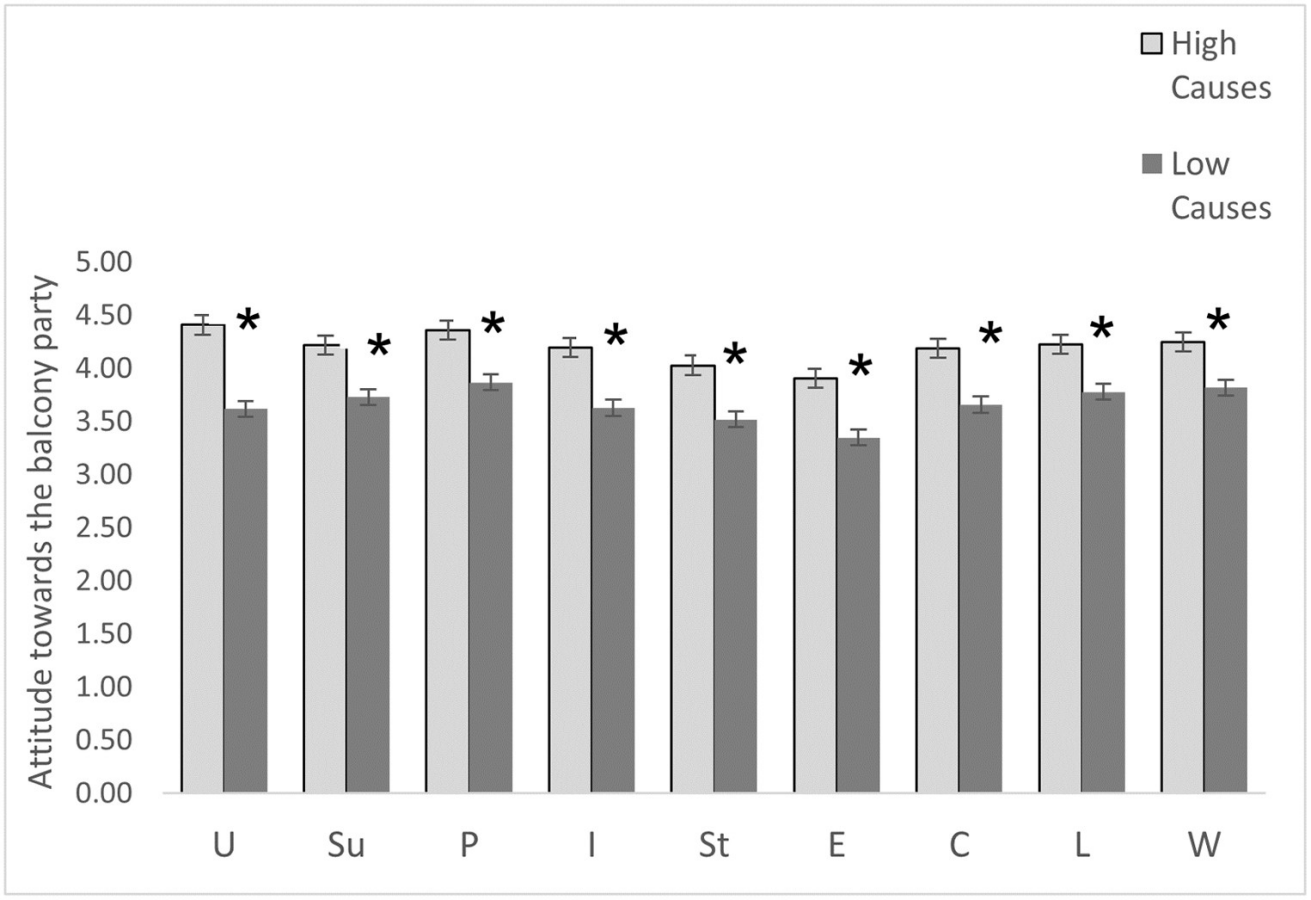
Average scores of attitude towards the balcony party divided into low levels of causes of SI and high levels of causes of SI groups. **U**–unity, **Su**-support, **P**-positive, **I**-isolation, **St**—stress, **E**-creating entertainment, **C**-connectedness, **L**-loneliness, **W**-worries. Note = * p < .05. The error bars represent the standard error.

The MANCOVA(with gender as a covariant) revealed a significant group cause effect (f(9,286) = 6.112, p = .000), suggesting an association between level of cause isolation and attitude towards the balcony parties. The cause level had a statistically significant group effect for balcony parties as inducing feelings of unity (f(1,294) = 48.677, p = .000), positive affect (f(1,294) = 14.857, p = .000), and support (f(1,294) = 16.252, p = .000), as well as reducing loneliness(f(1,294) = 14.096,p = .000), isolation (f(1,294) = 18.218, p = .000), worries (f(1,294) = 10.168, p = .002), and stress (f(1,294) = 10.928, p = .001). It also led to connectedness (f(1,294) = 12.844,p = .000) and entertainment (f(1,294) = 13.352, p = .000). As shown in Fig 2, examination of the means indicated that individuals with higher cause levels had a more positive attitude towards the balcony party.

#### Association between the self-reported levels of consequences of SI and attitude towards the balcony parties

MANCOVA (with gender, days in quarantine, education and country) revealed a non-significant main effect for the group (f(9,283) = .783, p = .632).

## Second part

### The beneficial outcomes of participating in parties

#### Association between self-reported levels of SI and beneficial outcomes of participating in the balcony parties

The MACNOVA (with days of quarantine) revealed a significant group effect (F(7,193) = 2.749 p = .010) that was qualified by marginally-significant type of party (balcony/pre-outbreak parties) by group interaction (F(7,193) = 1.961, p = .062). Type of measurement by group interaction was found significant for inducing feeling of unity (F(1,199) = 6.675, p = .010) and for reducing distress (f(1,199) = 10.711,p = .001), loneliness (f(1,199) = 9.278, p = .003) and isolation (f(1,199) = 7.505, p = .007). However, type of measurement by group interaction was found to be non-significant for inducing affect (f(1,199) = 1.495, p = .223), reducing worries (f(1,199) = 2.322, p = .129) and inducing connectedness (f(1,199) = 3.703,p = .056). As shown in Table 3, follow-up Mann–Whitney tests revealed significant group effects for pre-outbreak gatherings that became non-significant for parties during the outbreak. Examination of the means indicated that the beneficial outcomes associated with the balcony party were lower for the high SI group (e.g., unitedness from 4.18 during pre-outbreak parties to 3.94 during the balcony parties). However, the beneficial outcomes associated with the balcony party were lower for the SI group (e.g., unitedness from 3.85 during pre-outbreak parties to 4.07 during the balcony parties).

**Table 3:** The beneficial outcomes of participating in balcony parties vs. pre-COVID parties, according to the self-reported levels of SI and consequences of SI.

	Low SI	High SI	Statistical analyses
	Mean±s.e	mean±s.e	
Balcony parties:			
Unitedness	4.07±.089	3.94±.128	p = .837
Connectedness*	3.81±.091	4.07±.107	p = .028
Reduces isolation	4.06±.088	4.08±.111	P = .671
Reduces loneliness	4.03±.090	3.99±.116	p = .895
Reduces distress	3.61±.103	3.83±120	p = .148
Parties before COVID-19 outbreak			
Unitedness**	3.85±.083	4.18±.097	p = .002
Connectedness**	3.93±.098	4.36±.086	p = .002
Reduces isolation**	3.72±.101	4.24±.099	p = .000
Reduces loneliness**	3.75±.096	4.25±.088	p = .000
Reduces distress**	3.34±.11	4.09±.107	p = .000
	Low levels of consequences	High levels of consequences	Statistical analyses
	mean±s.e	mean±s.e	
Balcony parties:			
Unitedness	4.23±.091	3.89±.104	P = .142
Connectedness	3.89±.111	3.94±.089	P = .462
Reduces isolation	4.09±.104	4.05±.091	P = .901
Reduces loneliness	4.05±.115	3.99±.091	P = .632
Reduces distress	3.61±.132	3.76±.097	P = .372
Parties before COVID-19 outbreak			
Unitedness	3.91±.100	4.05±.083	P = .148
Connectedness*	3.89±.130	4.24±.077	p = .033
Reduces isolation**	3.66±.128	4.10±.087	P = .004
Reduces loneliness**	3.69±.122	4.12±.079	P = .003
Reduces distress**	3.30±.145	3.87±.095	P = .001

Note =

* p < .05,

** p < .01.

#### Association between the self-reported levels of causes of SI and beneficial outcomes of participating in the balcony parties

The MANCOVA (with gender.) revealed a significant group effect (F(7,193) = 2.667 p = .012) that was qualified by non-significant t party (balcony/pre-outbreak parties) by group interaction (F(7,193) = 1.188, p = .311).

#### Association between the self-reported levels of consequences of SI and beneficial outcomes of participating in the balcony parties

The MANCOVA (with days of quarantine and country) revealed a significant group effect (F(7,192) = 2.251 p = .032) that was qualified by non-significant type of party (balcony/pre-outbreak parties) by group interaction (F(7,192) = 1.750, p = .100). Type of party by group interaction was found significant for inducing feelings of unity (F(1,198) = 5.447, p = .021), connectedness (F(1,198) = 6.741, p = .010), and reducing isolation (F(1,198) = 4.864, p = .029), loneliness (F(1,198) = 7.185, p = .008) and anxiety (F(1,198) = 6.502, p = .012). However, type of party by group interaction was found to be non-significant for inducing affect (f(1,198) = 2.005, p = .158) and reducing worries (f(1,198) = 1.259, p = .263). As shown in Table 3, follow-up Mann-Whitney tests revealed significant group effects for pre-outbreak gatherings that became non-significant for parties during the outbreak. Examination of the means indicated that the beneficial outcomes associated with the balcony party were lower for the high levels of consequences group (e.g., unitedness from 4.05 during pre-outbreak parties to 3.89 during the balcony parties). However, the beneficial outcomes associated with the balcony party were higher for the low consequences group (e.g., unitedness from 3.91 during pre-outbreak parties to 4.23 during the balcony parties).

### The gap between attitude and beneficial outcomes of balcony parties

#### Association between the self-reported levels of SI and the gap between attitude and beneficial outcomes of balcony parties

**Fig 3:**
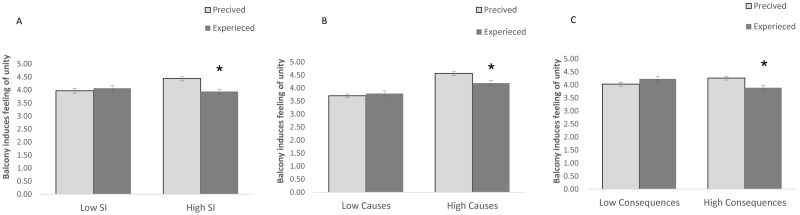
Average scores of attitude and beneficial outcomes of balcony parties divided into: (A) low and high SI groups, (B) low and high levels of causes of SI groups and (C) low and high levels of consequences of SI groups. Note = * p < .05, ** p < .01.

The MANCOVA (with days of quarantine) revealed a non-significant group effect (F(7,195) = 1.034, p = .409). However, there was a significant type of measurement (attitude/expectation towards the balcony parties vs. experience) by group interaction (F(7,195) = 3.363, p = .002), suggesting an association between the self-reported levels of SI and differences between the mean score of attitude towards balcony parties and the mean score of beneficial outcomes associated with participating in these parties. Type of measurement by group interaction was found to be significant for inducing feelings of unity with others (F(1,201) = 11.418, p = .001), but not for inducing feelings of worry (f(1,201) = 1.808,p = .180), anxiety (f(1,201) = .000, p = .988), isolation (f(1,201) = .676, p = .412, and loneliness (f(1,201) = .282, p = .596). Nor was it significant for inducing a sense of connectedness (f(1,201) = .044, p = .835) and the sense that balcony party was a great experience (f(1,201) = .012, p = .911). A Wilcoxon test revealed a significant effect for feeling of unity for individuals with high SI (p = .001), but not for individuals with low SI (p = .352). Examination of the means revealed that individuals with high levels of SI expected that the balcony party would evoke more of a feeling of unity than it actually did (see Fig 3A).

#### Association between the self-reported levels of causes of SI and the gap between attitude and beneficial outcomes of balcony parties

The MANCOVA (with gender) revealed a significant group effect (F(7,195) = 5.494, p = .000) that was qualified by a significant type of measurement (attitude/expectation towards the balcony parties vs. experience) by group interaction (F(7,195) = 2.588, p = .014), suggesting an association between the self-reported levels of causes of SI and the differences between the mean score of attitude towards balcony parties and the beneficial outcomes associated with participating in these parties. Type of measurement by group interaction was found to be significant for inducing feelings of unity with others (f(1,201) = 6.799, p = .010), but not for feelings of worry (f(1,201) = .167, p = .683), anxiety (f(1,201) = .964, p = .327), isolation (f(1,201) = .229, p = .632), and loneliness (f(1,201) = .192, p = .662). Nor was it significant for inducing a sense of connectedness (f(1,201) = .303, p = .583) and the sense that balcony party was a great experience (f(1,201) = .246, p = .620). A follow-up Wilcoxon revealed a significant effect for feelings of unity for individuals with high levels of causes of SI (p = .001), but not for individuals with low causes of SI (p = .352). Examination of the means revealed that individuals with high levels of causes of SI expected that the balcony party would evoke more of a feeling of unity than it actually did (see Fig 3B).

#### Association between the self-reported levels of consequences of SI and the gap between attitude and beneficial outcomes of balcony parties

The MANCOVA (with days of quarantine and country) revealed a non-significant consequences group effect (F(7,194) = .386, p = .910). However, there was a significant type of measurement (attitude/expectation towards the balcony parties vs. experience) by group interaction (F(7,194) = 2.360, p = .025), suggesting an association between the self-reported levels of consequences of SI and the differences between the mean score of attitude towards balcony parties and the mean score of beneficial outcomes associated with participating in these parties. Type of measurement by group interaction was found significant for inducing feelings of unity with others (F(1,201) = 7.772, p = .006), but not for inducing a sense that the balcony was a great experience (f(1,201) = .738, p = .391), connectedness (f(1,201) = .019, p = .891), loneliness (f(1,201) = .195, p = .659), isolation (f(1,201) = .359, p = .551), anxiety (f(1,201) = .000, p = .992), and worry (f(1,201) = 2.863, p = .092). A follow-up Wilcoxon test revealed a significant effect for feeling of unity for individuals with high levels of consequences of SI (p = .003), but not for individuals with low levels of consequences of SI (p = .098). Examination of the means revealed that individuals with high levels of consequences of SI expected that the balcony party would evoke more of a feeling of unity than it actually did (see Fig 3C).

## Discussion

With the new realities of social isolation and quarantine, videos on social media of citizens taking to their balconies to clap, sing, and dance to boost morale are growing increasingly common. To our knowledge, this is the first research to look at balcony parties as a novel way to mitigate the psychological costs of social isolation. While technology-mediated forms of communication are a critical component of modern life, especially during the coronavirus outbreak, they lack basic elements of naturalistic face-to-face social interactions, such as sharing the same physical space [18]. Here, we propose the possibility that balcony parties enabled, at least to a certain extent, for more naturalistic social interactions in which different forms of social alignment were induced [20–22]. This may explain why these public community balcony gatherings involve neighbors aligning their behavior with each other through hand-clapping, singing, and dancing. Importantly, as these various forms of social alignment are expected to lead to rewarding sensations [23], we speculated that individuals with higher levels of social isolation will embrace this rewarding sensation, thus expressing a more positive attitude towards balcony parties. Our findings confirmed this hypothesis by showing that participants who experienced high levels of social isolation during the outbreak of COVID-19 perceived the balcony parties as more beneficial in comparison to those who experienced low levels of social isolation. Further analysis revealed that this association was significant only for the causes of SI, but not for the consequences of SI, suggesting that those who missed face-to-face social and naturalistic interaction with their friends and family (i.e. causes of SI) were those who perceived the balcony parties as more beneficial in comparison to similar events that preceded the COVID-19 outbreak. The opposite pattern was observed when individuals were asked about their participation in these parties. Those with high levels of SI experienced balcony parties as less beneficial than similar events that preceded the COVID-19 outbreak. In contrast, individuals with low levels of SI showed an opposing pattern. Further analysis revealed that this association was significant only for the consequences of SI, but not for the causes of SI, suggesting that those who felt socially isolated, lonely, and anxious were those who perceived the balcony parties as less beneficial in comparison to similar events that preceded the COVID-19 outbreak. Finally, for high SI participants, balcony parties did not satisfy their expectation of creating a feeling of unity with others. However, for their low SI counterparts, balcony parties did meet their expectations. Further analysis revealed that this association was significant both for the consequences of SI and for the causes of SI. This suggests that balcony parties did not satisfy the expectation of creating a feeling of unity with others, for participants who felt higher levels of causes of SI and higher levels of consequences of SI.

In a changing globalized society, new epidemic outbreaks may result in more periodic episodes of social distancing and quarantine. In turn, this may lead to higher and more widespread levels of social isolation and loneliness [27], a major public health issue which can exert a significant impact on physical and mental wellbeing [28]. As such, health professionals need to develop novel solutions to decrease the psychological cost of social isolation. However, coping directly with loneliness and social isolation by strengthening social support or by forming social networks [29] is impeded by the situational factor–the demand to maintain social distance and stay-at-home regulations. Additionally, nostalgia has been suggested as an alternative coping strategy to deal with loneliness. Nostalgia refers to an individual’s sentimental longing for the past [30]. It is considered to be inherent in human nature, resulting from evocation of emotions when reflecting fondly on positive past memories [31]. As noted earlier, our findings show that those with high levels of SI reflected on pre-outbreak gatherings in the past as more beneficial than the balcony parties of the present. Moreover, this tendency was not evident among individuals with low levels of social isolation. This may suggest that higher levels of social isolation are associated with increased tendency to augment subjective perceptions of social support by drawing on nostalgic memories. In other words, individuals with high levels of SI may employ nostalgia as a psychological resource, and this was manifested in their tendency to reflect on pre-outbreak gatherings of the past as more beneficial than the balcony parties of the present. This interpretation, however, requires caution, with further research needed to rule out other potential explanations such as differences in clinical characteristics between the two groups.

Resilience, which refers to the ability to recover from shock, insult, or disturbance [32], is also of great relevance for examining the psychological cost of social isolation during the COVID-19 outbreak. Interestingly, it has been found that highly resilient individuals are most likely to recruit nostalgia in response to loneliness [31]. While our findings suggest the possibility that nostalgia acts as a strategy to respond to emotional challenges associated with social isolation, the question of whether nostalgia can boost levels of resilience to the cost of social isolation deserves to be investigated.

Our findings reveal a gap between how individuals perceived the balcony parties and how they actually experience it. This suggests the possibility that the expectation for rewarding sensations associated with participating in balcony parties was not fulfilled. Indeed, a within-subjects approach revealed that individuals with high levels of SI expected the balcony parties to induce more feeling of unity with others than they actually experienced. Importantly, the discrepancy between expectation and actual experience was not observed for individuals with low levels of SI–their experience of feeling united with others did meet their expectations. The discrepancy between how the balcony parties were perceived and how they were actually experienced may be interpreted in view of the predictive coding perspective [33]. In this framework, the brain is essentially a “prediction machine” with the ultimate goal of “prediction error” minimization; that is, the discrepancy between incoming information and generated predictions [34]. As such, it is possible that the experience of individuals with higher SI during the balcony parties was different from their experiential prediction, with this gap constituting a “prediction error”. Since prediction error is experienced as an unpleasant state, the brain constantly strives to minimize this error [35]. For this, there are two options: 1) through action to change the environment to fulfill expectations and 2) by optimizing these expectations for better sensation matching. Achieving alignment with others has been argued to reduce prediction error by making the environment more predictable [35, 36]. Previous work has shown that social isolation can modulate the way in which individuals perceive and mirror the expressions and actions of others [37]. Hence, it is reasonable to propose that those with high levels of social isolation SI suffer from deficiencies in their capacity to achieve alignment during the balcony parties, with this constituting a prediction error.

It should be noted that this study has several limitations. First, although the number of respondents was relatively high, it is possible that it is not a representative sample of the entire group of people under quarantine, especially since respondents were mostly women. Second, respondents required access to a computer to respond, which suggests a self-selection effect may have occurred. More specifically, it suggests that those who agreed to participate may have been more educated, younger and had higher socioeconomic status than the overall group who were quarantined (in Israel and in Italy). Third, all data was derived from self-report questionnaires which is vulnerable to a recall bias. In this context, it is important to mention that we strived to obtain as much information about the adverse effects of quarantine as close to the event as possible so as to eliminate recall bias. Hence, the project was initiated when concerns about COVIID-19 were still a part of daily life in both in Israel and Italy. In fact, during this period of time, Israel and Italy were more or less under lockdown. Hence, although the interval between the last party attended and when the testing occurred was not measured, we believe that testing occurred as close to the event as possible. Fourth, the cross-sectional nature of this study limits our ability to determine any causality in the results. Fifth, the questions that were asked about the attitude towards the balcony party were mostly formulated to assess what balcony parties do in general, whereas the questions that were asked about the beneficial outcomes of these parties were asked about how these parties affected the individual. One may argue that attitude measures that look primarily at 3rd person perspectives may have little or nothing to do with what they think the benefit would be/was for them. However, this limitation could not be avoided, since the questionnaire assessed attitudes towards the balcony party that was filled out by individuals who did not participate in balcony parties. For these individuals, questions asked about attitudes towards the balcony party from 1st person perspectives are irrelevant. Finally, although we controlled for the variability associated with important potential confounders, by including these factors as covariates in the analyses we did not measure all potential confounders that could affect the group differences (e.g., individual psychiatric history). It is important to note, however, that the study was based on a relatively large sample and was conducted during the pandemic (between March 23th, 2020 and April 2th, 2020) while balcony parties were growing in popularity. We, therefore, believe that despite its limitations, the current study offers important information on the psychological impact of COVID-19.

In conclusion, due to globalization and urban population density, social isolation may emerge as a prominent new reality in an age of pandemics. The costs of social distancing and quarantine thus need to be closely evaluated in the context of psychological suffering. As such, balcony parties constitute a novel counter-measure to provide a measure of naturalistic face-to-face social interaction, thereby mitigating the psychological costs of social isolation. Our findings suggest that its role is dependent on the self-reported levels of SI. Balcony parties benefited individuals with lower SI and in particular those with lower consequences of SI, whereas the opposite was observed for high SI participants, and in particular those with high levels of consequences of SI. Earlier research has linked feelings of closeness with neighbors and intention to take a vaccine or wash hands more frequently during a pandemic [38]. Future empirical efforts should consider the possibility that participating in balcony parties is beneficial for those with low levels of SI, not only to reduce the emotional cost of social isolation but also to increase precautionary habits needed during the coronavirus, in particular washing hands.

As a final note, in earlier studies exposure to a virus was found to be necessary, but not sufficient, to cause illness. Notably, chronic stress and social isolation, among other psychological factors, may partly determine development of an acute infectious respiratory illness [39]. In line with this, our findings on assessing mitigation cost strategies of social isolation may be valuable in reducing the risk of developing COVID-19.

## Supporting information

S1 Table Descriptive statistics and results of comparison between demographic data of respondents to the first part of the survey and respondents to the second part of the survey. (DOCX)

S2 Table Correlation analysis of scales of the questionnaire “Psychological Impact of Quarantine and Social Isolation”. (DOCX)

S1 File (XLSX)
