# Supplementary material for: A link between social isolation during the coronavirus outbreak and social alignment in balcony parties
Source: PLoS One. 2022 Apr 6;17(4):e0264109. doi: 10.1371/journal.pone.0264109 (PMC8985989; doi:10.1371/journal.pone.0264109)
Supplement: S1 Table — (DOCX) [file pone.0264109.s001.docx]

**Table S1: Descriptive statistics and results of comparison between demographic data of respondents to the first part of the survey and respondents to the second part of the survey**

| **Demographic data** | **Total** | **Both parts**  **N = 207** | **First part only**  **N = 96** | **Statistical**  **Analyses** |
| --- | --- | --- | --- | --- |
| Parametric measures [mean ± S.D.]  Age* | 27 | 26 | 31 | U=7051, p =.000 |
| Days in Quarantine* | 14 | 14 | 18 | U= 8232 ,p=.035 |
| Non-parametric measures[Number (%)]    Gender (male/female)*  Education(Middle School, High School, Bacelor, Master, PhD or higher)*  *  Family Status (marriage/single)  How many children in the family (no children, 1 children, 2-4 childrens)  Country(europ/israel)* | 57(18.8%), 246(81.2%)  12(4%),99(32.7%),79(26.1%),105(34.7%)8(2.6%)  202(66.7%),100(33%  227(74.9%), 32(10.6%), 43(14.2%),1(0.3%)  108(35.6%),195(64.4%) | 30(14.2%),181(85.8%)  8(3.8%),82(38.9%),52(24.6%),63(29.9%),6(2.8%)  68(32.2%), 142(67.3%)  157(74.9%),23(10.9%), 31(14.7%),0  58(27.5%),153(72.5%) | 27(29.3%),65(70.7%)  4(4.3%),17(18.5%),27(29.3%),42(45.7%),2(2.2%)  32(34.8%), 60(65.2%)  70(76.1%),9(9.8%) 12(13%),1(1.1%)  50(54.3%)42(45.7%) | X² = 9.6, p = .002  X² = 13.46 p = .009  X² =.604, p = .739  X² = .2.516, p = .472  X²=20.149, p=.000 |

Note= * p<.05
