# Supplementary material for: A link between social isolation during the coronavirus outbreak and social alignment in balcony parties
Source: PLoS One. 2022 Apr 6;17(4):e0264109. doi: 10.1371/journal.pone.0264109 (PMC8985989; doi:10.1371/journal.pone.0264109)
Supplement: S2 Table — (DOCX) [file pone.0264109.s002.docx]

|  | L | D | I | F-C-F | F |
| --- | --- | --- | --- | --- | --- |
| L | 1 |  |  |  |  |
| D | .454^**^ | 1 |  |  |  |
| I | .311^**^ | .273^**^ | 1 |  |  |
| F-C-F | .262^**^ | .337^**^ | .334^**^ | 1 |  |
| F | .193^**^ | .311^**^ | .304^**^ | .819^**^ | 1 |

**Table S2: Correlation analysis of scales of the questionnaire “Psychological Impact of Quarantine and Social Isolation”**

L–"Since the corona outbreak I feel lonely;" D-"Since the corona outbreak I feel distress;" I-"Since the corona outbreak I feel more socially isolated; " F-T-F–" Since the corona outbreak I miss face-to-face social interaction; "F-"Since the corona outbreak I miss naturalistic interaction with my friends/family".
